# Virtual Cell and Metabolic Control Analysis: Control Coefficients for Glycolytic Flux Are Highly Dependent on the Subsystem Selected for Analysis

**DOI:** 10.3390/life16030414

**Published:** 2026-03-04

**Authors:** Michael V. Martinov, Fazoil I. Ataullakhanov, Eugene S. Protasov, Victor M. Vitvitsky

**Affiliations:** 1 Center for Theoretical Problems of Physico-Chemical Pharmacology, Russian Academy of Sciences, Moscow 109029, Russia; martinov.michael@gmail.com (M.V.M.); ataullakhanov.fazly@gmail.com (F.I.A.); protasov_evgenii@mail.ru (E.S.P.); 2 Dmitriy Rogachev National Medical Research Center for Pediatric Hematology, Oncology, and Immunology, Ministry of Healthcare of the Russian Federation, Moscow 117198, Russia

**Keywords:** systems biology, metabolic control analysis, control coefficients, glycolysis, energy metabolism, ion homeostasis

## Abstract

The metabolic control analysis (MCA) was applied to several subsystems selected from the model of human erythrocyte energy metabolism. These subsystems represent varying degrees of simplification of energy metabolism, from the simplest subsystem of the first three glycolytic reactions that determine the steady-state rate of glycolysis, to an expanded subsystem that includes all glycolytic reactions plus passive and active ion transport across the cell membrane. The control coefficients of enzyme activities for the rate of glycolysis are found to be very different in different subsystems. However, no specific trend is observed in changes in control coefficients as the subsystem becomes more complex. Thus, in subsystems containing only glycolysis, the control coefficients of hexokinase (HK) and phosphofructokinase (PFK) together amount to 0.99. When ATPases are added, this value decreases to 0.18 and below, and the maximum control coefficient goes to ATPase (0.82–1.00). It would seem that there is a natural decrease in the contribution of HK and PFK to the regulation of the rate of glycolysis as the dimension of the system increases. However, disabling the allosteric regulation of PFK by AMP completely changes the picture. In a subsystem containing only glycolysis, disabling this regulation does not affect the control coefficients. After adding ATPase to such a subsystem, the HK and PFK control coefficients increase, and the control coefficient of ATPase takes on a negative value. Thus, we found that in extended subsystems involving glycolysis and ATPase or transmembrane ion transport, information on the initial regulation of glycolysis may not be revealed in the MCA results. It appears that the MCA alone cannot reveal regulatory mechanisms of metabolic systems in the presence of strong allosteric and feedback regulation.

## 1. Introduction

Over the past two decades, a vast amount of biochemical information has been accumulated thanks to the development of various “omics” technologies. However, it remains questionable to what extent this has expanded our knowledge of the regulation of cellular metabolic processes. We are still very far from understanding how multiple metabolic processes in the cell interact to ensure their coordinated functioning, which in turn maintains cellular homeostasis and physiology. Understanding the regulation of metabolic networks in cells is impossible without a systems approach to cell biology, including mathematical modeling of metabolic pathways.

In systems biology, a number of approaches have been developed for studying large and complex metabolic systems (networks). These include metabolic control analysis, biochemical system analysis (power-law approximation), and various versions of flux analysis. However, the results of these analyses are highly dependent on the structure, size, and complexity of the metabolic systems [1,2,3,4,5,6,7]. One of the main approaches to the analysis of the regulation of metabolic pathways in systems biology is the metabolic control analysis (MCA) [4,5,6]. This theoretical framework has been developed to elucidate in quantitative terms to what extent the various reactions of metabolic pathways determine the fluxes and metabolite concentrations. The MCA uses control coefficients to describe effects of parameters on steady-state system properties such as metabolic flow rate. Mathematically, all the control coefficients represent the fractional change in a system property, dX/X, in response to a fractional change in a parameter dP/P, in the limit as dP tends to zero:

(1) $$C=\frac{dX}{dP}\cdot \frac{P}{X}\;=\frac{dlnX}{dlnP}$$

This theory may allow us to identify the stages in a metabolic system that have the most significant impact on the metabolic flow in the system. The theory has been used to analyze metabolic regulation in human erythrocyte glycolysis [8,9], in muscle glycolysis [10], in cancer cell glycolysis [11], in the oxidative phosphorylation pathway [12], etc. The literature describes attempts to obtain control coefficients in glycolysis using experimental changes in the activity of all glycolytic enzymes [13].

How reliable are the results obtained using MCA? Naturally, one of the conditions for the reliability of the results is the use of correct mathematical models. However, the absolute correctness of models is always questionable, since we may not know all the regulatory links in the modeled system.

In this work, we tested the robustness of the MCA results using simple theoretical objects. To this end, we applied MCA to analyze the regulation of glycolytic flux in several relatively simple model systems with well-studied regulation. These systems were selected as subsystems of a “virtual cell,” a mathematical model of the human erythrocyte. This model includes glycolysis, adenine nucleotide turnover, and ATPases, including Na/K-ATPase transport. It also describes transmembrane ion transport and osmotic regulation of cell volume [14]. This model was successfully used to explain the effect of glycolytic enzyme deficiency on the viability and functional integrity of human erythrocytes [14]. These subsystems were obtained from the main model by simplifying it to different levels and include the glycolytic pathway at constant [ATP], [ADP], and [AMP]; the glycolytic pathway plus ATPase; and the glycolytic pathway plus passive and active ion transport across the cell membrane. A detailed description of all subsystems considered in this work is presented in the Materials and Methods and in Supplementary Materials sections. These subsystems were used as theoretical objects of research in which we know and understand all the regulatory mechanisms.

We found that MCA indicates a transfer of maximal control coefficients from hexokinase and phosphofructokinase in the subsystems that include glycolysis only to ATPase in the subsystem that includes ATP consumption and further to the permeability of the cell membrane for cations in the subsystem with transmembrane ion transport. Thus, information about the initial regulation of the rate of glycolysis by hexokinase and phosphofructokinase may be lost during the transition to subsystems with ATP consumption. However, the transfer of the main control coefficients from hexokinase and phosphofructokinase to ATPase does not occur in the absence of allosteric activation of phosphofructokinase by AMP. Thus, the same extension of the system under study may or may not radically change the distribution of control coefficients. It appears that the MCA alone cannot reveal regulatory mechanisms of metabolic systems in the presence of strong allosteric and feedback regulation.

## 2. Materials and Methods

### 2.1. Mathematical Model Description

**Figure 1:**
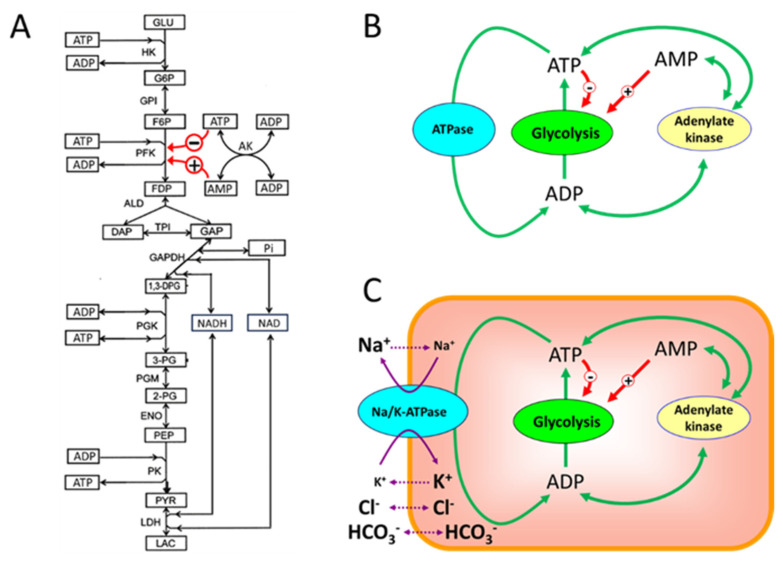
The main subsystems selected from the virtual cell for MCA. (**A**)—biochemical reactions included in the first subsystem (glycolysis) for the MCA; (**B**)—the cell energy metabolism; (**C**)—the model of a cell that maintains a non-equilibrium distribution of Na^+^ and K^+^ between the cytoplasm and the environment. The red arrows show allosteric activation (+) and inhibition (−) of the PFK reaction by ATP and AMP. The green arrows show the interconversions between ATP, ADP, and AMP. The solid and dotted purple arrows show active and passive ion fluxes through the cell membrane, respectively. The ion symbol size inside and outside the cell is proportional to the ion concentration. Abbreviations used in the paper are described in Table 1.

As it was mentioned above, the virtual cell was constructed to explain a dependence of human erythrocyte viability and functional integrity on activities of glycolytic enzymes [14]. The virtual cell includes glycolysis, adenylate kinase equilibrium, synthesis and degradation of adenine nucleotides (ATP, ADP, and AMP), transport Na/K-ATPase, additional ATPase, not associated in the model with any specific function, passive transmembrane transport of Na^+^, K^+^, Cl^−^, and HCO_3_^−^ ions, and osmotic cell volume regulation [14]. The main model (the virtual cell) was constructed based on an experimentally validated model of glycolysis in human erythrocytes [15]. The main model explained why hemolytic anemias occur with certain deficiencies in glycolytic enzyme activity [14]. Thus, this model was validated against a large pool of experimental data. Later, modified quantitative models of glycolysis in human erythrocytes were constructed based on the main model, which provided a good description of the experimental data [16]. This can also be considered a validation of the model. In this study, three main subsystems were selected from the main model (virtual cell). The first subsystem (subsystem (1)) includes the glycolytic pathway at constant levels of [ATP], [ADP], and [AMP] (Figure 1A). The second subsystem (subsystem (2)) combines the glycolytic pathway with adenylate kinase equilibrium and an ATPase whose reaction rate depends linearly on [ATP] (energy metabolism, Figure 1B). The third subsystem (subsystem (3)) adds passive and active ion transport across the cell membrane to the glycolytic pathway and adenylate kinase equilibrium (Figure 1C). Four additional subsystems were developed on the background of these three main subsystems. They included subsystem (1a) obtained from subsystem (1) by truncation of the lower part of the glycolysis (the enzymatic reactions after phosphofructokinase); subsystem (1b) obtained from subsystem (1) by removing the allosteric regulation by AMP from the equation for the reaction rate of PFK; subsystem (2a) obtained from subsystem (2) by replacing the linear ATPase with the hyperbolic dependence of the reaction rate on [ATP]; and subsystem (2b) obtained by adding to subsystem (1b) an ATPase with a linear dependence of the reaction rate on [ATP].

All subsystems include adenylate kinase equilibrium $K_{eq}^{AK}=\frac{\left[ATP\right]\left[AMP\right]}{{\left[ADP\right]}^{2}}=1.$ Reactions of the 2,3-diphosphoglycerate shunt, which bypasses the ATP-producing PGK reaction, were excluded from the subsystems. Concentrations of glucose, orthophosphate, pyruvate, lactate, the sum of [ATP], [ADP], and [AMP] (pool of adenine nucleotides), and the sum of [NAD] and [NADH] are considered constant and equal to 5 mM, 1 mM, 70 µM, 1.2 mM, 1.745 mM, and 50 µM, respectively.

The rate of ATP production in glycolysis in our models (except for subsystem (1a), which is described separately) is equal to the following sum of reaction rates: V_PK_ + V_PGK_ − V_HK_ − V_PFK_. Here V_HK_, V_PFK_, V_PGK_, and V_PK_ denote the rates of HK, PFK, PGK, and PK reactions, respectively. According to the stoichiometry of glycolysis (Figure 1A), after the aldolase reaction, the steady-state glycolytic flux increases twofold. Thus, the steady-state rate of ATP production in our models is twice the rate of the HK and PFK reactions and the rate of glucose consumption. In all subsystems, the normal physiological steady-state rates of glucose consumption and ATP production are equal to 1.12 mM/h and 2.24 mM/h, respectively, at a normal ATP concentration of 1.47 mM. Below are more detailed descriptions of the subsystems.

**Table 1:** Abbreviations used in the paper.

1,3-DPG	1,3-diphosphoglycerate
2-PG	2-phosphoglycerate
3-PG	3-phosphoglycerate
AK	adenylatekinase
ALD	aldolase
Ani	the sum of intracellular concentrations of anions penetrating the cell membrane ([Cl^−^] + [HCO_3_^−^])
DAP	dihydroxyacetone phosphate
ENO	enolase
F6P	fructose-6-phosphate
FDP	fructosediphosphate
G6P	glucose-6-phosphate
GAP	glyceraldehyde phosphate
GAPDH	glyceraldehydephosphate dehydrogenase
GLU	glucose
GPI	glucose-6-phosphate isomerase
HK	hexokinase
LAC	lactate
LDH	lactate dehydrogenase
MCA	metabolic control analysis
PEP	phosphoenolpyruvate
PFK	phosphofructokinase
PGK	phosphoglycerate kinase
PGM	phosphoglycerate mutase
Pi	phosphate inorganic
PK	pyruvate kinase
PYR	pyruvate
Perm	relative non-selective cell membrane permeability for cations
Pump	Na/K-Pump (Na/K-ATPase)
TPI	triosephosphate isomerase

#### 2.1.1. Subsystem (1)

The first subsystem includes glycolysis at constant concentrations of ATP, ADP, and AMP equal to 1.47, 0.237, and 0.038 mM, respectively. The ratio between these concentrations corresponds to the equilibrium of the adenylate kinase reaction, and their absolute values correspond to the normal physiological state in human erythrocytes [14]. The description of glycolysis in the model includes all eleven enzymatic reactions shown in Figure 1A. The equations for the reaction rates and parameter values were taken from [14].

#### 2.1.2. Subsystem (1a)

This subsystem (truncated glycolysis) was obtained from subsystem (1) by removing the enzymatic reactions from aldolase to lactate dehydrogenase. Concentrations of glucose, orthophosphate, and the sum of [ATP], [ADP], and [AMP] in this model were the same as in the first subsystem. Concentrations of pyruvate, lactate, and the sum of [NAD] and [NADH] make no sense in this subsystem. The rate of ATP production in this subsystem is equal to the rate of PFK reaction multiplied by two. The normal physiological steady-state rates of glucose consumption and ATP production in this subsystem are the same as in subsystem (1).

#### 2.1.3. Subsystem (1b)

This subsystem is the same as subsystem (1), except that the allosteric regulation (activation) of PFK by AMP was eliminated from the equation for the PFK reaction rate. The original equation for the rate of the PFK reaction is as follows [14]:

(2) $$V_{PFK}=A_{PFK}\frac{1.1\left[ATP\right][F6P]\left(\frac{1}{1+\left[AMP\right]/K_{PFK}^{3}}+\frac{2[AMP]}{K_{PFK}^{3}+[AMP]}\right)}{\left(K_{PFK}^{2}+[ATP]\right)\left(K_{PFK}^{1}+[F6P]\right)\left(1+10^{8}\frac{{\left(1+\left[ATP\right]/K_{PFK}^{4}\right)}^{4}}{{\left(1+\left[AMP\right]/K_{PFK}^{3}\right)}^{4}{\left(1+\left[F6P\right]/K_{PFK}^{5}\right)}^{4}}\right)}$$

The parameters in this equation have the following values: $A_{PFK}=380$ mM/h, $K_{PFK}^{1}=0.1$ mM, $K_{PFK}^{2}=2$ mM, $K_{PFK}^{3}=0.01$ mM, $K_{PFK}^{4}=0.195$ mM, and $K_{PFK}^{5}=3.7\cdot 10^{-4}$ mM.

In this equation, the AMP concentration was replaced by a constant value of 0.038 mM that is equal to the physiologically normal AMP concentration in human erythrocytes [14]. All other parameters were the same as in subsystem (1). This subsystem was considered to test how changes in enzyme kinetics can affect the results of MCA without changes in the subsystem dimension and complexity.

#### 2.1.4. Subsystem (2)

In this subsystem, the glycolysis (subsystem (1)) was supplemented with ATPase with the linear dependence of the reaction rate on [ATP]:

(3) V^L^_ATPase_ = α^L^_ATPase_[ATP]

At normal physiological conditions the ATPase activity α^L^_ATPase_ is equal to 1.524 h^−1^ that provides the steady-state ATP consumption rate equal to the rate of ATP production in glycolysis (2.24 mM/h).

In this subsystem, which represents cell energy metabolism, [ATP], [ADP], and [AMP] are variables. The dynamics of the [ATP], [ADP], and [AMP] are determined from the balance of production and consumption of ATP and ADP under the assumption that the adenylate kinase reaction is in equilibrium and the sum of the concentrations of adenine nucleotides is constant:

(4) $$\frac{de}{dt}=-V_{HK}-V_{PFK}+V_{PGK}+V_{PK}-V_{ATPase}$$

(5) $$K_{eq}^{AK}=\frac{\left[ATP\right]\left[AMP\right]}{{\left[ADP\right]}^{2}}=1$$

(6) $$\left[ATP\right]+\left[ADP\right]+\left[AMP\right]=1.744\;mM$$

(7) e = 2[ATP] + [ADP]

Here, V_HK_, V_PFK_, V_PGK_, V_PK_, and V_ATPase_ denote the rates of HK, PFK, PGK, PK and ATPase reactions, respectively. $K_{eq}^{AK}$ denotes the equilibrium constant of the AK reaction.

#### 2.1.5. Subsystem (2a)

This subsystem was obtained from subsystem (2) by replacing the ATPase with a linear dependence of the reaction rate on [ATP] with an ATPase with a hyperbolic dependence of the reaction rate on [ATP]:

(8) $${V^{H}}_{ATPase}={\alpha^{H}}_{ATPase}\frac{\left[ATP\right]}{\left[ATP\right]+K_{ATPase}}$$

The value of the Michaelis constant for ATP in this equation was equal to 10 μM. At this value of K_ATPase_, the rate of the ATPase reaction is independent of [ATP] at physiological levels of [ATP] in the model. Under normal conditions, the ATPase activity α^H^_ATPase_ is equal to 2.255 mM/h. At this parameter value the normal steady-state rate of ATP consumption is equal to the normal rate of ATP production in glycolysis.

#### 2.1.6. Subsystem (2b)

This subsystem includes glycolysis with disabled allosteric regulation of phosphofructokinase by AMP (subsystem (1b)), and ATPase with a linear dependence of the reaction rate on ATP. The ATPase activity (α^L^_ATPase_) value in this subsystem is the same as in subsystem (2). All other parameters have the same values as in subsystem (1b).

#### 2.1.7. Subsystem (3)

In subsystem (3), the model was extended compared with subsystem (2) by the addition of the Na^+^, K^+^, Cl^−^, and HCO_3_^−^ transport across the cell membrane. Transmembrane transport of Na^+^ and K^+^ is both passive and active, whereas for anions that penetrate the cell membrane (Cl^−^ and HCO_3_^−^), transport is only passive. The passive transmembrane ion fluxes are described in Goldman approximation, taking into account the electroneutrality of the cell content and transmembrane potential [14]. The normal permeability of the cell membrane for Na^+^ (G_Na0_) and for K^+^ (G_K0_) in the model is 0.0422 and 0.0424 1/h, respectively. At these values of cell membrane permeability, the model ensures an ATP consumption rate equal to the normal steady-state rate of ATP production in glycolysis (2.24 mmol/h). We assume a non-selective increase or decrease in the permeability of the cell membrane for cations as follows:

(9) G_Na_ = G_Na0_ + Δ; G_K_ = G_K0_ + Δ

Here, Δ denotes a value of a change in the cell membrane permeability for cations.

We also introduced the parameter Perm corresponding to the relative permeability of the cell membrane for cations:

(10) $$Perm=\frac{G_{Na}}{G_{Na0}}\approx \frac{G_{K}}{G_{K0}}$$

The validity of this equation follows from the fact that G_Na0_ ≈ G_K0._

We assume that there is an equilibrium distribution of penetrating anions between the cytoplasm and the environment due to the high permeability of the cell membrane for these anions. Extracellular concentrations of Na^+^, K^+^, and the sum of extracellular concentrations of anions penetrating the cell membrane (Cl^−^ and HCO_3_^−^) are constant and equal to 145, 5, and 150 mM, respectively [14]. The non-functional ATPase of subsystem (2) was replaced by the Na/K-ATPase (Na/K-pump), which transfers three Na^+^ ions from the cytoplasm to the medium in exchange for the transfer of two K^+^ ions from the medium to the cytoplasm through the hydrolysis of one ATP molecule. The rate of ATP consumption by the Na/K pump is described by the following equation [14]:

(11) V_pump_ = α_pump_[Na^+^][ATP]

At normal conditions parameter α_pump_ is equal to 0.152 mM/h.

With the chosen parameter values, the normal steady-state rates of glucose consumption and ATP production in this model are 1.12 and 2.24 mM/h, respectively.

The cell volume is considered constant.

The models of all subsystems are systems of ordinary differential equations. These equations describe the kinetics of glycolytic metabolite concentrations and other variables, such as adenine nucleotides and Na+ and K+ ions. Additionally, algebraic equations describe the conservation of cofactor pools and equilibrium reactions. The right-hand sides of the differential equations contain the sum of the rates of accumulation and consumption of the corresponding metabolites/variables.

Additional information on the subsystems is provided in the Supplementary Materials Section (Equations (S1)–(S41)).

### 2.2. Mathematical Model Analysis

The CVODE (v7.2) library was used to calculate the kinetics of the models [17]. Steady states were determined using the KINSOL (v7.2) library [17]. The steady-state rate of glycolysis (the glycolytic flux) was determined as the steady-state rate of the PK reaction (as the steady-state rate of the PFK reaction multiplied by two in subsystem (1a)). Thus, the glycolytic flux is equal to the rate of ATP production in glycolysis. To calculate the control coefficients, we increased and decreased the enzyme activity or the cell membrane permeability to cations by 0.1% compared to the initial value and calculated the corresponding steady states of the model and the corresponding glycolytic flux values. The control coefficients for the glycolytic flux when changing these parameters were calculated using the following formula:

(12) $$C=\frac{f\left(p+\Delta p\right)-f\left(p-\Delta p\right)}{2\Delta p}\cdot \frac{p}{f(p)}$$

Here, p is the initial value of the parameter, and ∆p = 0.001p. f(p), f(p + Δp), and f(p − Δp) are the steady-state values of the glycolytic flux at the initial value of the parameter (p) and with an increase and the decrease in the parameter value by Δp, respectively. The dependence of the steady state of the model on the parameters was investigated using the AUTO2000 package [18].

Modular metabolic control analysis (metabolic supply–demand analysis) [19,20,21,22] of subsystem (2) was performed using MATLAB (v9.12) software [23]. During the analysis, the steady-state dependence of the ATP production rate in the subsystem on ATP concentration, taken as a parameter, was obtained using the MATLAB fsolve() function [23]. Using this dependence and the dependence of the ATP consumption rate on [ATP], the steady state of the subsystem was determined. For this steady state, elasticity values were calculated for the ATP production and ATP consumption modules. Flux and concentration control coefficients were calculated from the elasticity values according to [20].

The basal steady state of glycolysis is the same in all subsystems. To assess the deviation of glycolytic reactions from equilibrium, mass-action ratios were calculated for each reaction using the steady-state concentrations of metabolites obtained in subsystem (1). The known value of the equilibrium constant of the reaction was divided by the corresponding mass-action ratio, and the resulting quotient (R) was used as a measure of the deviation of the reaction from equilibrium:

(13) $$R=\frac{K_{eq}}{\prod P_{j}/\prod S_{j}}$$

Here K_eq_ denotes the equilibrium constant of the reaction, and P_j_ and S_j_ denote the reaction products and substrates, respectively. At equilibrium, R is equal to one and greater than one if the reaction proceeds in the direction of glycolytic flow.

The values of equilibrium constants for glycolytic reactions were taken from [14] with the exception of the HK, PFK, and PK reactions. The values of equilibrium constants for these reactions were taken from [24].

The source files of the models can be downloaded from https://doi.org/10.5281/zenodo.18753989, (accessed on 24 February 2026).

## 3. Results

### 3.1. Control Coefficients for Glycolytic Flux in Different Subsystems

**Figure 2:**
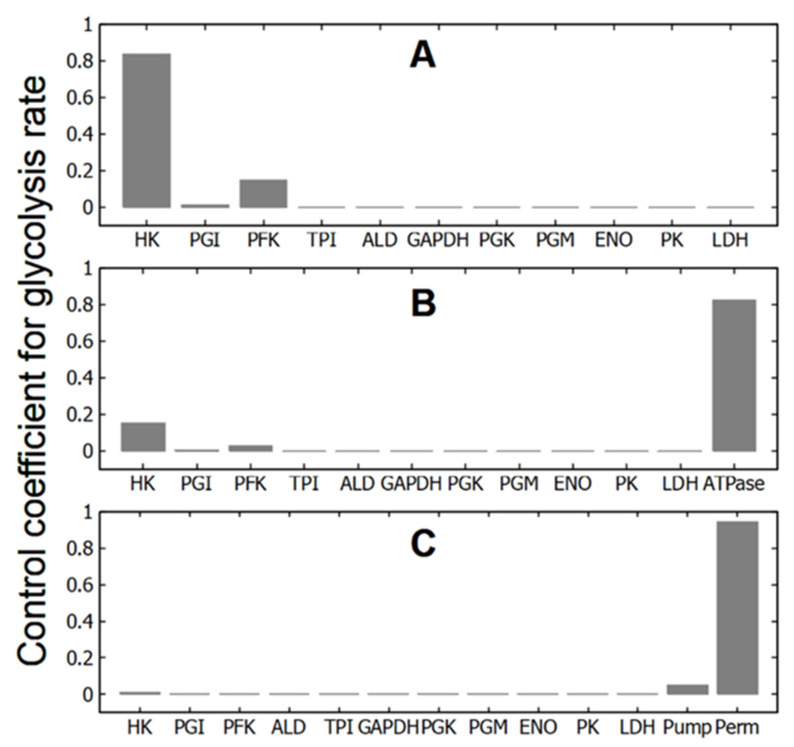
Control coefficient values of glycolytic enzymes, ATPase, Na/K-pump, and cell membrane permeability for cations for glycolytic flux in different subsystems selected from the virtual cell. (**A**)—glycolysis at constant concentrations of adenine nucleotides (subsystem (1)); (**B**)—glycolysis with ATPase (subsystem (2)); (**C**)—glycolysis with passive transmembrane ion transport and Na/K-pump (Na/K-ATPase) (subsystem (3)).

In the subsystems, which include only glycolysis at constant concentrations of ATP, ADP and AMP (subsystems (1), (1a), and (1b)), HK and PFK have the largest control coefficients for the glycolytic flux (Figure 2A, Table 2). The control coefficients of other enzymes are negligible. Hexokinase has the lowest activity of the glycolytic enzymes [14], and it is not surprising that changes in its activity have a significant impact on the glycolytic flux and thus on the rate of ATP production in glycolysis. And PFK is the main regulatory enzyme of glycolysis, which determines the glycolytic flux and the rate of ATP production in response to changes in the concentration of adenine nucleotides, citrate, NH_4_^+^, pH, etc. [25,26,27,28]. Thus, the MCA confirms that in these subsystems the regulation of glycolytic flux is produced by HK and PFK, in accordance with much experimental and theoretical data [11,15,26,27,29,30].

It is interesting to note that a significant simplification of subsystem (1) by removing all enzymes of the lower part of glycolysis (subsystem (1a)) did not affect the values of the control coefficients in any way (Table 2). This is consistent with the idea that the steady-state glycolytic flux is determined (regulated) by the enzymes of the upper part of glycolysis (HK, GPI and PFK). Also, the exclusion of allosteric regulation of PFK by AMP does not affect the control coefficients (subsystem 1b, Table 2). Thus, neither a significant change in the system dimension (the transition between subsystems (1) and (1a)) nor a change in the kinetics of the reaction rate of one of the key enzymes in the system (the transition from subsystem (1) to subsystem (1b)) within glycolysis affects the values of the control coefficients in these subsystems.

At the same time, a relatively small change in the system dimension by adding a linear ATPase (subsystem (2)) leads to a sharp change in the values of the control coefficients (Figure 2B, Table 2). The control coefficients of the HK and PFK decrease several times, the control coefficients of other glycolytic enzymes remain negligible, and the maximum control coefficient switches to ATPase activity. In the case of a hyperbolic dependence of the rate of ATP consumption on [ATP], the decrease in the control coefficients of HK and PFK and the increase in the control coefficient of ATPase are stronger than in the case of a linear ATPase (Table 2). However, the effect does not change fundamentally. At the same time, the addition of ATPase to the subsystem with disabled allosteric regulation of PFK by AMP (subsystem (2b)) does not lead to a decrease in the control coefficients of HK and PFK. They even increase (Table 2). In this case, the control coefficient of ATPase takes on a rather large, but negative value (Table 2). Thus, adding ATP consumption to glycolysis may or may not result in significant changes in the control coefficients of HK and PFK for glycolysis.

The results obtained indicate that in subsystems (2) and (2a), the activation of ATP consumption causes an increase in glycolysis rate. Contrary to this, in subsystem (2b) the rate of glycolysis, and consequently the rate of ATP production, decreases when ATP consumption is activated.

Finally, in subsystem (3) that includes passive and active transmembrane ion transport and in which all ATP consumption is associated with the active transport of Na^+^ and K^+^ across the cell membrane (Na/K-ATPase), the biggest control coefficient is associated with the permeability of the cell membrane to cations (Figure 2C, Table 2). The control coefficient of Na/K-ATPase (Na/K-pump) is significantly smaller, while the control coefficients of HK and PFK become negligible (Figure 2C, Table 2). Thus, a change in the subsystem due to the association of ATPase activity with the performance of a certain function (in our case, with active transmembrane transport of cations) leads to the transfer of control coefficients from HK, PFK and ATPase to the parameter regulating this function (the permeability of the cell membrane to cations).

Please note that for all subsystems the sum of the control coefficients is very close to 1. This is consistent with the MCA summation theorem [4,5,6]. The deviations of the sums of control coefficients from 1 in Table 2 do not exceed 0.05% and are most likely due to the errors in our calculations.

We would like to note that the ATP production control coefficients in all subsystems were absolutely the same as for the glycolytic flux.

The results discussed above show that, under normal allosteric regulation of PFK by AMP, the addition of ATP consumption processes to glycolysis leads to a redistribution of control coefficients. This applies to both non-specific ATP consumption and ATP consumption associated with transmembrane ion transport. In these cases, control is transferred from glycolytic enzymes to ATPase and to the permeability of the cell membrane to Na^+^ and K^+^ ions. However, neither ATPase nor cell membrane permeability directly controls glycolytic flux. At the same time, the low control coefficients of glycolytic enzymes indicate a loss of apparent control within glycolysis itself. This suggests that the regulation of glycolytic flux is no longer carried out primarily by glycolytic enzymes. Below we will try to consider how the regulation of glycolytic flow is carried out in subsystems containing ATPase or ion transport. We will also try to understand whether the original regulation of glycolysis is preserved in these systems.

### 3.2. Mechanisms of the Glycolytic Flux Regulation: The Role of HK and PFK

**Figure 3:**
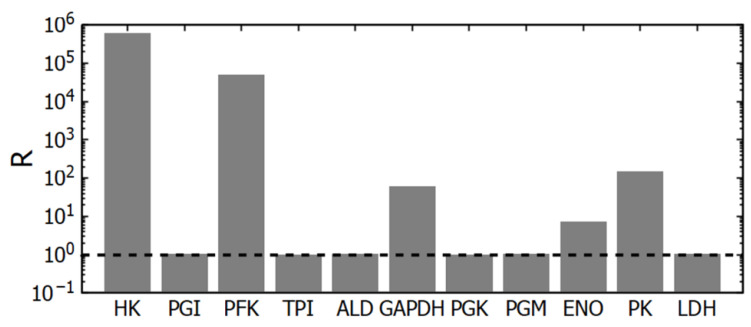
Deviation of glycolytic reactions from equilibrium (R). At R = 1 (the position is shown by the dashed line), the reaction is at equilibrium. For the PGI, TPI, ALD, PGK, PGM, and LDH reactions, the R value lies within the range of 1.004–1.04. The mass-action ratios were calculated using the steady-state concentrations of metabolites obtained in subsystem (1).

Our data obtained using subsystem (1) (simply glycolysis) confirm that glycolytic flux is regulated by the interaction of HK and PFK [15,26,27,29]. The corresponding reactions are the farthest from thermodynamic equilibrium among glycolytic reactions and are practically irreversible (Figure 3). There is one more irreversible step in glycolysis—the PK reaction. However, PK cannot regulate the steady-state glycolytic flux. Indeed, an increase in PK activity should lead to a decrease in the concentration of its substrate, PEP, and all other glycolytic intermediates between PK and PFK. But the PFK reaction is irreversible and insensitive to all these metabolites, including its product (FDP). Thus, [PEP] will decrease until the PK reaction rate decreases to the initial level equal to the steady-state glycolytic flux determined by HK and PFK. Similarly, a decrease in PK activity should lead to an increase in [PEP] until it stimulates the PK reaction rate to the initial level of the steady-state glycolytic flux, determined by HK and PFK. Of course, it works until PK activity does not decrease so low as to make PK a rate-limiting enzyme in glycolysis. At that point, the steady state in metabolism disappears. Thus, changes in PK activity definitely affect the steady-state metabolite concentrations but do not affect the steady-state glycolytic flux. It is confirmed by a small control coefficient for PK (Figure 2A). An important role of PK in glycolysis is that it prevents pyruvate and lactate from interfering with upstream reactions.

The GAPDH reaction is also relatively far from equilibrium (Figure 3). Regarding this reaction, one can say that under the intracellular conditions the reaction is reversible and has no allosteric regulation. The reaction rate is proportional to the concentrations of substrates and products and a change in GAPDH activity will cause just rearrangement in its substrates and products concentrations that brings its rate equal to the glycolytic flux determined by HK and PFK. Thus, despite being relatively far from equilibrium, this reaction cannot regulate the steady-state glycolytic flux.

The PGI, TPI, ALD, PGK, PGM, and LDH reactions are close to thermodynamic equilibrium. For these reactions, the ratio of the equilibrium constant to the corresponding mass-action ratio in the model (the R value) lies in the range 1.004–1.04 (Figure 3). The enzymes that catalyze these reactions have relatively high activity [14]. Therefore, even near the state of equilibrium, these reactions can provide normal glycolytic flux.

**Figure 4:**
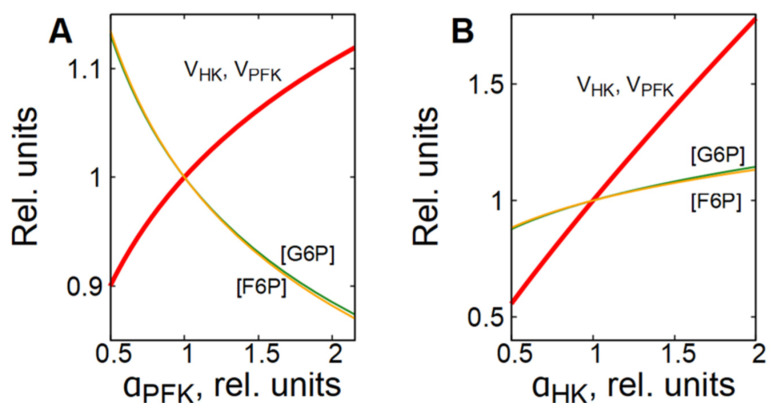
Dependence of steady-state concentrations of metabolites and rates of enzymatic reactions in subsystem (1) on the activity of PFK (**A**) and HK (**B**). All values are normalized to normal physiological values of the corresponding parameters and variables.

As it was mentioned above, the PFK is the main enzyme that regulates the glycolytic flux in cells. The PFK reaction rate is sensitive to changes in concentration of different metabolites, including adenine nucleotides, citrate, NH_4_^+^, etc. [25,26,27,28]. However, to provide a steady-state glycolytic flux, the rates of the preceding reactions must be adjusted to be equal to the PFK reaction rate. In most cells in which glycolysis is not coupled to glycogen metabolism, this is achieved due to a feedback regulation of the HK reaction rate via G6P, which is a strong inhibitor of HK [15,26,27,29]. Activation of PFK causes a decrease in [G6P] and an acceleration of the HK reaction, whereas inhibition of PFK causes an increase in [G6P] and an inhibition of the HK reaction (Figure 4A).

This is why we note here that the glycolytic flux is regulated by the interaction of phosphofructokinase and hexokinase. Activation or inhibition of HK causes a corresponding increase or decrease in [F6P], the PFK substrate and activator, which in turn, causes acceleration or inhibition of the PFK reaction (Figure 4B).

**Figure 5:**
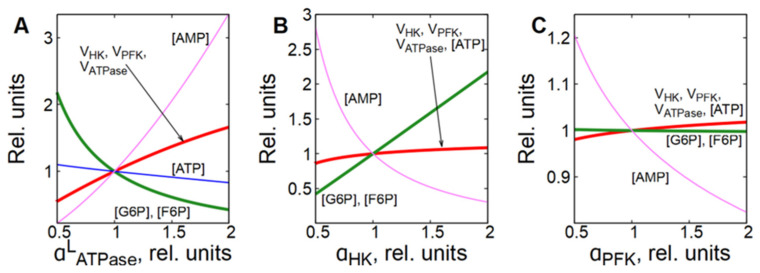
Dependence of steady-state concentrations of metabolites and rates of enzymatic reactions in subsystem (2) on the activity of ATPase (**A**), HK (**B**) and PFK (**C**). All values are normalized to normal physiological values of the corresponding parameters and variables.

**Figure 6:**
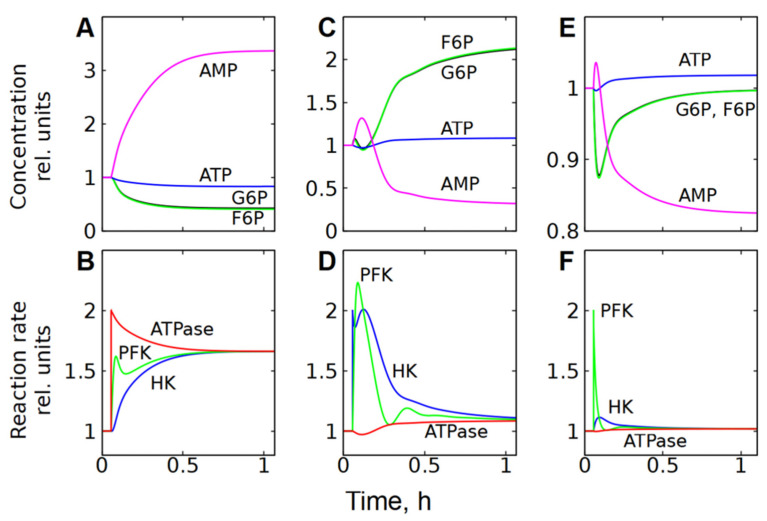
Kinetics of metabolite concentrations and enzymatic reaction rates in subsystem (2), representing cell energy metabolism, after a twofold increase in ATPase activity (**A**,**B**); HK activity (**C**,**D**); and PFK activity (**E**,**F**). The parameter was changed at a time equal to 0.056 h.

In subsystem (2), which includes glycolysis and ATPase, the MCA shows that ATPase has the biggest control coefficient for glycolytic flux (Figure 2B). On the other hand, ATPase itself has no direct effect on the rate of glycolysis. The influence of ATPase on glycolytic flux is transmitted through the concentrations of ATP and AMP. Indeed, activation of ATPase causes a decrease in [ATP] and accumulation of AMP in the cell, which activates the PFK reaction (Figure 5A and Figure 6A,B). The acceleration of the PFK reaction causes a decrease in [F6P] and [G6P], which, in turn, leads to the acceleration of the HK reaction because G6P is a strong inhibitor of HK (Figure 5A and Figure 6A,B). Simultaneously, the rate of ATPase reaction decreases due to the decrease in [ATP] (Figure 6A,B). Eventually, HK and PFK establish a new steady-state glycolytic flux corresponding to the new ATP consumption rate (Figure 5A and Figure 6A,B). The described mechanism represents a negative feedback regulation of glycolysis by [ATP].

However, this subsystem resists changes in the steady-state glycolytic flux even in the case of significant changes in HK or PFK activity if ATPase activity does not change (Figure 5B,C). For example, a twofold increase in HK activity causes a slight increase in [ATP], a significant decrease in [AMP], and an accumulation of G6P and F6P (Figure 5B and Figure 6C). Ultimately, the accumulation of one PFK activator (F6P) and the depletion of the other (AMP) compensate for each other and return the PFK reaction rate to almost the original level (Figure 6D). At the same time, the accumulation of G6P, an inhibitor of HK, reduces the rate of the HK reaction and also brings it almost to the initial level. Eventually, both reactions reach the same steady-state rate, which is about 10% higher than the initial rate (Figure 6D). This rate corresponds to the rate of the ATPase reaction, which increases slightly due to the increase in [ATP] (Figure 6C,D).

A twofold increase in the activity of PFK causes a decrease in the concentrations of its activators—F6P and AMP, a slight increase in [ATP], and a significant decrease in [AMP] (Figure 5C and Figure 6E). The decrease in [AMP], which is an activator of FPK, compensates for the acceleration of the FPK reaction caused by the increase in its activity (Figure 6F). The HK reaction rate slightly increases due to the decrease in [G6P]. Eventually, both reactions reach the same rate, which is only a few percent higher than the initial rate (Figure 6F). As with HK activation, this rate corresponds to the rate of ATPase reaction, which increases slightly due to the increase in [ATP] (Figure 5C and Figure 6E,F).

**Figure 7:**
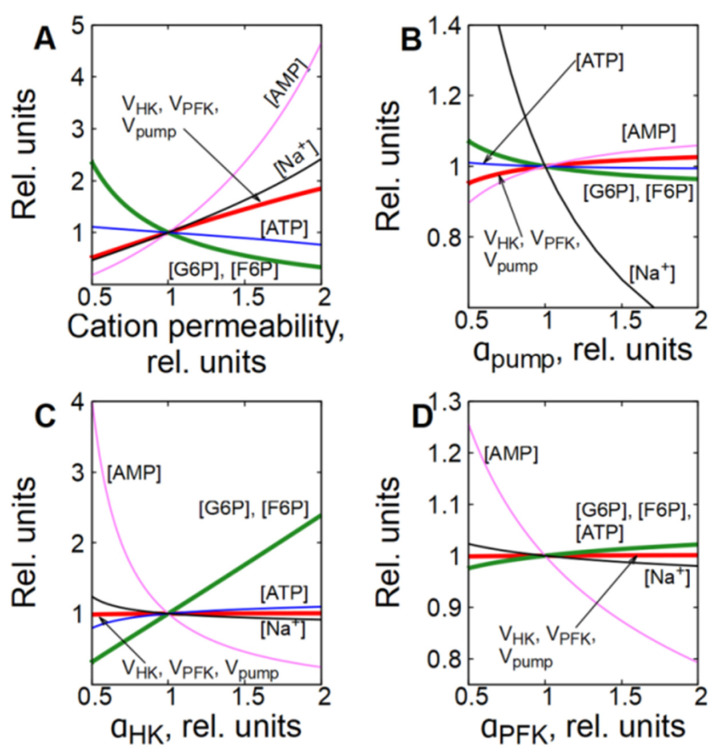
Dependence of steady-state concentrations of metabolites, [Na^+^], and rates of enzymatic reactions in subsystem (3) on the permeability of the cell membrane to cations (**A**), on the activity of Na/K-ATPase (**B**), HK (**C**) and PFK (**D**). All values are normalized to normal physiological values of the corresponding parameters and variables. When the relative permeability of the cell membrane to cations changes from 0.5 to 2.0 (**B**), the relative concentration of Na^+^ ions changes from 1.9 to 0.5.

**Figure 8:**
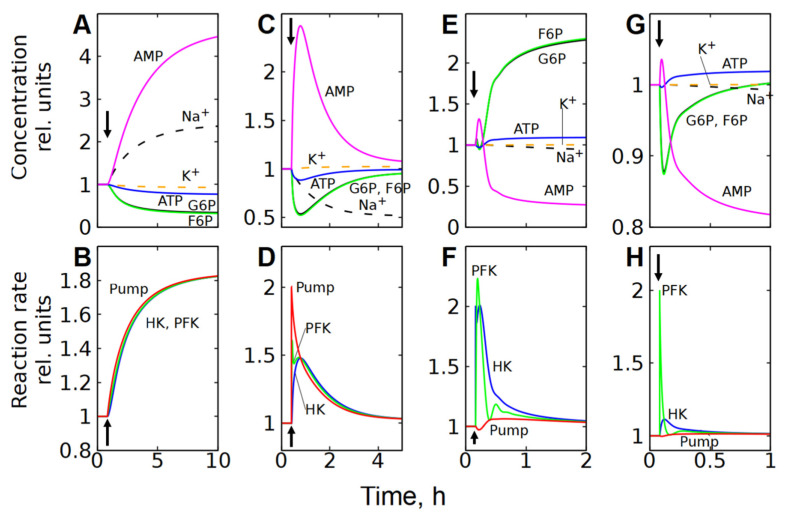
Kinetics of metabolite concentrations, [Na^+^], [K^+^], and enzymatic reaction rates in subsystem (3), which includes the transmembrane ion transport, after a twofold increase in the permeability of the cell membrane to cations (**A**,**B**), and a twofold increase in Na/K-ATPase (**C**,**D**), HK (**E**,**F**), and PFK (**G**,**H**) activity. Kinetics of [Na^+^] and [K^+^] are shown by the dashed lines. The parameter changed at the time indicated by the vertical arrow.

In subsystem (3), which includes transmembrane ion transport and a functional ATPase (Na/K-pump), the permeability of the cell membrane to cations has the biggest control coefficient for the glycolytic flux (Figure 2C). Figure 7A and Figure 8A,B show that an increase in the permeability of the cell membrane to cations causes an accumulation of Na^+^ ions in the cytoplasm, which leads to an increase in the Na/K-ATPase rate, since it is proportional to [Na^+^] (Equation (11)). An increase in the rate of ATP consumption causes an increase in glycolytic flux in the same way as in subsystem (2) with an increase in ATPase activity. It is evident that the changes in the concentration of metabolites caused by a twofold increase in the permeability of the cell membrane for cations in the subsystem (3) (Figure 7A and Figure 8A) are similar to the changes in the concentration of metabolites caused by a twofold increase in ATPase activity in subsystem (2) (Figure 5A and Figure 6A). Thus, changes in the permeability of the cell membrane to cations affect the rate of ATP consumption, which affects the concentration of ATP and AMP. This, in turn, affects the rates of the PFK and HK reactions, which determine the steady-state glycolytic flux corresponding to the rate of ATP consumption.

However, unlike subsystem (2), an increase in the Na/K-ATPase activity alone in subsystem (3) does not cause a significant activation of glycolysis (Figure 7B and Figure 8C). This occurs because an increase in Na/K-ATPase activity causes a decrease in [Na^+^] in the cytoplasm (Figure 7B and Figure 8C). Since the rate of the Na/K-ATPase reaction is proportional to [Na^+^], this results in a decrease in the reaction rate (Figure 7B and Figure 8C). As a result, the initial increase in the Na/K-ATPase reaction rate due to the increase in its activity is almost completely compensated by the decrease in the reaction rate caused by the decrease in the concentration of [Na^+^] in the cytoplasm. A decrease in cytoplasmic [Na^+^] results in an increase in passive Na^+^ influx due to an increase in the transmembrane ion gradient. Thus, the rate of the Na/K-ATPase reaction and the glycolytic flux increase only to compensate for the increased passive Na^+^ influx. Finally, the steady-state reaction rates of Na/K-ATPase, HK, and PFK increase only by a few percent.

An increase in HK or PFK activity in subsystem (3) has only a minor effect on glycolytic flux, and changes in metabolite concentrations and reaction rates (Figure 7C,D and Figure 8E–H) are similar to those observed in the second subsystem (Figure 5B,C and Figure 6C–F), which can be explained by the same mechanisms as for subsystem (2).

Please note that in energy metabolism, the characteristic time to reach a new steady state is 1–2 h (Figure 6 and Figure 8E–H). In the ion homeostasis system, this time is significantly longer, reaching 5–10 h (Figure 8A–D).

In Figure 6 and Figure 8, the rates of enzymatic reactions after changing the parameters converge to the same final steady-state value in relative coordinates. Please note, however, that according to glycolysis stoichiometry (Figure 1A) the steady-state rate of ATP production in glycolysis is equal to twice the rate of the HK or PFK reaction. Thus, in absolute coordinates, the rates of the HK and PFK reactions and the rates of ATP consumption converge to different levels.

The above discussion was conducted in the case of increasing key parameters in the subsystems. A similar discussion can be conducted in the case of decreasing these parameters.

The steady-state metabolite profiles obtained in three different subsystems at twofold increase and a twofold decrease in parameters (HK, PFK, GPI, PGK, ATPase, and Na/K-ATPase activities and the permeability of the cell membrane to cations) are shown in the Supplemental Materials (Figures S1–S3).

The conducted model study of subsystems (2) and (3) shows that the initial regulation of the rate of glycolysis in them is preserved, and the rate of glycolysis is determined by the rates of HK and PFK reactions. In this case, both ATPase and the permeability of the cell membrane to cations regulate the rate of glycolysis due to the allosteric regulation of the rate of the PFK reaction by ATP and AMP. However, in these cases, information on the initial regulation of glycolysis is not revealed in the MCA results.

### 3.3. Modular Metabolic Control Analysis (MMCA) of the Subsystem Including Glycolysis and ATPase (Subsystem (2))

We attempted to overcome the limitations of classical metabolic control analysis (MCA) and to obtain more detailed information on the interaction between glycolysis and linear ATPase. For this purpose, we used an approach based on dividing metabolism into modules of production and consumption of an intermediate metabolite. In different studies, this approach has been referred to as modular analysis [19], modular metabolic control analysis (MMCA) [21], metabolic supply–demand analysis [20], generalized supply–demand analysis (GSDA) [22], and others. This approach was developed to extend infinitesimal MCA and make it applicable to real metabolic systems with large changes in variables. Accordingly, it is expected to provide a more adequate analysis of metabolic regulation in realistic systems. Within MMCA, flux control coefficients are calculated not for individual reactions, but for entire pathways (modules).

**Figure 9:**
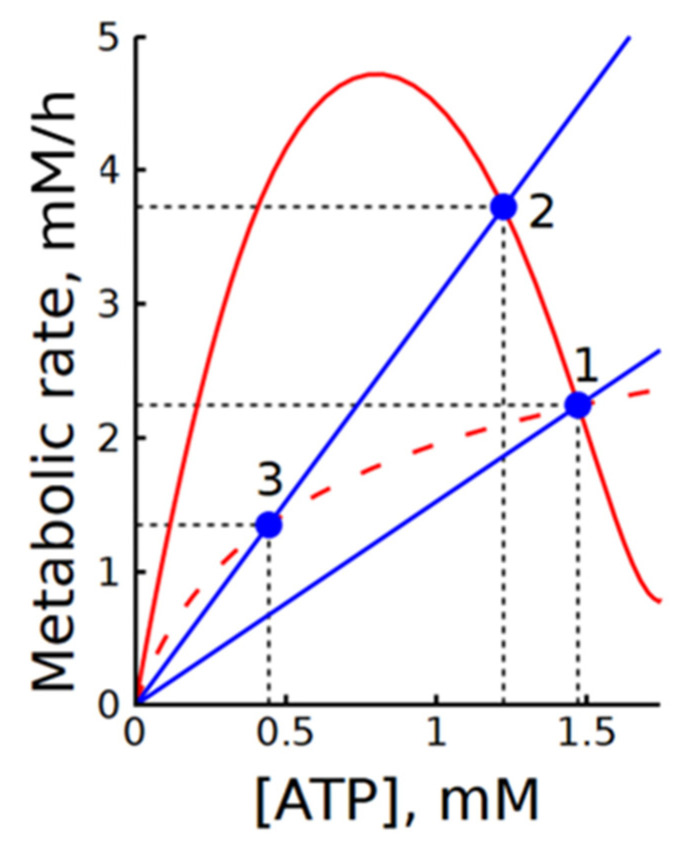
Illustration of the interaction between steady-state dependencies of ATP production and consumption rates on [ATP] in subsystems (2) and (2b). Solid and dashed red lines represent the steady-state dependencies of ATP production rate on [ATP] in subsystems (2) and (2b), respectively. Blue lines show the dependencies of ATP consumption rate on [ATP] at the normal physiological steady state (point 1) and at a twofold increase in ATPase activity (points 2 and 3). Blue circles indicate steady states. Black dashed lines indicate steady-state levels of metabolic rates and ATP concentrations. Note that the steady-state dependencies of ATP production rate on [ATP] in subsystems (1) and (1b) overlap with the solid and dashed red lines, respectively.

In our case, subsystem (2) is divided into modules of ATP production (glycolysis) and ATP consumption (ATPase), with ATP acting as an intermediate metabolite. For clarity, Figure 9 shows the steady-state dependencies of ATP production and consumption rates on ATP concentration. In subsystem (2), the dependence of the ATP production rate on [ATP] has a bell-shaped form with a steep descending branch in the range of physiologically normal [ATP] values. The slope of the ATP consumption rate dependence on [ATP] corresponds to ATPase activity. The intersections of these graphs define the steady states of the subsystem.

At the physiologically normal value of ATPase activity, MMCA yields the following values for the elasticities in the steady state of subsystem (2): $\varepsilon_{ATP}^{p}=\frac{[ATP]\cdot dV_{p}}{V_{p}d[ATP]}=-4.57$, $\varepsilon_{ATP}^{c}=\frac{[ATP]\cdot dV_{c}}{V_{c}d[ATP]}=0.99$, and the corresponding flux control coefficient for ATP production and ATP consumption modules are: $C_{p}^{J}=\frac{\varepsilon_{ATP}^{c}}{\varepsilon_{ATP}^{c}-\varepsilon_{ATP}^{p}}=0.18$, and $C_{c}^{J}=\frac{-\varepsilon_{ATP}^{p}}{\varepsilon_{ATP}^{c}-\varepsilon_{ATP}^{p}}=0.82$.

Here, the indices p and c denote ATP production and consumption, respectively.

The concentration control coefficients for ATP production and ATP consumption modules are: $C_{p}^{ATP}=0.18$ and $C_{c}^{ATP}=-0.18$.

The large negative value of the elasticity coefficient $\varepsilon_{ATP}^{p}$ reflects the steep slope of the descending branch of the ATP production rate dependence on [ATP]. The much larger flux control coefficient of the ATP consumption module compared to the production module indicates that the glycolytic flux in this subsystem is mainly determined by ATP consumption. This result agrees well with the data shown in Figure 2. As seen in Figure 9, activation of ATPase leads to an almost equivalent increase in the steady-state rate of glycolysis. At the same time, the change in ATP concentration is small because of the steep slope of the descending branch.

The results show that MMCA itself, similar to classical MCA, does not provide direct information on the regulation of glycolysis in subsystem (2). It looks like a much more informative approach is the analysis of the plots showing ATP production and consumption rates as functions of [ATP]. The steady-state dependence of glycolysis rate on [ATP] does not depend on the presence or absence of ATPase. Thus, it is clear that the intrinsic regulation of glycolytic flux in this subsystem is preserved. The addition of ATPase merely imposes an additional constraint that prevents the system from deviating strongly from the steady state. Comparison of the graphs in Figure 9 allows estimation of the degree of ATP concentration stabilization upon changes in ATPase activity. Moreover, it makes it possible to determine the range of ATPase activity changes over which glycolytic regulation can ensure an increase in ATP production in accordance with increased ATP consumption. This range is determined by the maximum in the steady-state dependence of the glycolysis rate on the ATP concentration.

Interestingly, when allosteric activation by AMP is removed from the phosphofructokinase rate equation (Equation (2) and Equation (S28)), the steady-state dependence of glycolysis rate on [ATP] becomes monotonically increasing (Figure 9). In this case, ATPase activation leads to a decrease in the steady-state glycolysis rate (Figure 9). This explains the negative control coefficient of ATPase in subsystem 2b (Table 2).

## 4. Discussion

Our results show that in different subsystems selected from the same initial model, MCA points to different steps controlling the rate of glycolysis (Figure 2, Table 2). In subsystems that include only glycolysis with constant concentrations of [ATP], [ADP], and [AMP] (subsystems (1), (1a), and (1b)), HK and PFK have the maximum control coefficients for the rate of glycolysis. Moreover, the values of the coefficients are practically independent of the subsystem dimension and the presence of specific allosteric regulation of the PFK by AMP (Table 2). In subsystems including glycolysis and ATPase, the maximum control coefficient shifts from HK and PFK to ATPase activity in the presence of allosteric regulation of PFK by AMP (subsystems (2) and (2a)). In the absence of allosteric regulation of PFK by AMP (subsystem (2b)), HK and PFK retain high values of the control coefficients, and the ATPase control coefficient takes on a negative value (Table 2). Finally, in the subsystem that includes a functional ATPase coupled with active transmembrane transport of cations (subsystem (3)), the maximum control coefficient shifts from HK, PFK, and ATPase to the permeability of the cell membrane to cations (Figure 2C, Table 2).

It should be noted that the dependence of the rate of glycolysis on the activity of ATP consumption or on the permeability of the cell membrane to cations (which determines the rate of active transport of cations) corresponds to the main task of ATP-producing systems—to ensure the rate of ATP production is equivalent to the rate of its consumption. In other words, the rate of ATP production (the rate of glycolysis) in cells should increase in response to an increase in the rate of ATP consumption. For example, a similar response of the rate of glycolysis to the rate of ATP consumption and the permeability of the cell membrane to cations has been demonstrated in erythrocytes in a number of experiments [15,31,32,33]. From this perspective, the regulation of glycolysis obtained in subsystem (2b) (a decrease in the rate of glycolysis upon activation of ATP consumption) appears biochemically and physiologically meaningless. The increase in the rate of glycolysis upon activation of ATP consumption in eukaryotic cells occurs due to the allosteric regulation of PFK by ATP and AMP [15,26,27,29]. Exclusion of allosteric regulation by AMP from the equation for the rate of the PFK reaction disrupts the physiologically meaningful regulation of the rate of glycolysis that is confirmed here using MCA. It is interesting to note that a similar meaningless result (negative control coefficient of ATPase) was previously obtained in a model of yeast glycolysis that did not take into account the allosteric regulation of PFK by AMP and ATP [34].

Thus, in the presence of correct allosteric regulation of PFK by ATP and AMP, MCA reveals a physiologically meaningful dependence of the rate of glycolysis on the activity of ATP-consuming processes in the cell or on the permeability of the cell membrane for cations. But MCA does not reveal the mechanism of this regulation. As it was mentioned above, neither ATPase nor cell membrane permeability directly controls glycolytic flux. At the same time, low control coefficients of glycolytic enzymes in subsystems (2), (2a), and (3) indicate that regulation of the glycolytic flux cannot be carried out within glycolysis itself. However, a detailed model analysis of subsystems (2) and (3) performed in this work shows that the internal regulation of glycolysis by HK and PFK is maintained in these subsystems and the rate of glycolysis in them is determined by the interaction of PFK and HK, which depends on the allosteric regulation of PFK by ATP and AMP (Figure 3, Figure 4, Figure 5, Figure 6, Figure 7 and Figure 8). As a result, it can be concluded that in the process of MCA, information about the internal regulation of glycolysis in subsystems (2) and (3) is lost.

This loss of information can be explained from the point of view of glycolysis flux regulation described above. As one can see from the analysis of subsystems (2) and (3), the glycolytic flux is adjusted due to allosteric regulation of PFK and HK to provide the ATP production rate equal to the rate of ATP consumption determined by ATPase activity or by cell membrane permeability to cations (Figure 6 and Figure 8). This regulation is quite perfect and maintains the required rate of glycolysis even with significant variations in glycolysis parameters, including variations in the activities of glycolytic enzymes (Figure 5 and Figure 7). Since the control coefficients in the MCA reflect the effect of changes in enzyme activity on the glycolytic flux, the illusion is created that the rate of glycolysis is not regulated by PFK and HK; that is, there is a loss of information about the internal regulation of glycolysis.

Impaired allosteric regulation of PFK does not affect the results of the MCA when applied only to glycolysis (subsystem (1b)). However, the regulation of glycolytic flux is impaired. As a result, in a system that includes ATPase (subsystem (2b)) MCA gives a physiologically meaningless result (a negative control coefficient of ATPase). In such a system (in contrast to subsystems (2) and (2a)) the rate of glycolysis depends significantly on the activities of PFK and HK and the control coefficients of PFK and HK remain high (Table 2). The negative control coefficient is not unusual from a metabolic point of view. It appears, for instance, when the rate of metabolite production shows a hyperbolic dependence on its concentration, whereas the rate of its consumption depends linearly on concentration. This situation is exactly what occurs in subsystem (2b) (Figure 9). The intersection of the production and consumption graphs determines the steady-state metabolite concentration and the equal rates of its production and consumption. Under these conditions, an increase in the slope of the metabolite consumption graph (i.e., activation of metabolite consumption) shifts the intersection point (steady state) toward lower metabolite concentrations and metabolic rates (Figure 9). Thus, under these conditions, the metabolic rate decreases as the activity of metabolite consumption increases. This behavior is described by a negative control coefficient of metabolite consumption activity for the metabolite production flux. However, this creates a problem in the context of energy metabolism, because it predicts that an increase in the activity of ATP-consuming processes should lead to a decrease in both the rate of ATP production and [ATP]. Such a metabolic response contradicts the basic logic of energy metabolism. According to this logic, the rate of ATP production should increase in response to increased ATP consumption.

Our data show that even when studying simple metabolic systems with fully known and understood regulation, MCA can produce contradictory and confusing results. At the same time, information about the regulation of metabolic pathways included in these systems may be lost. A full understanding of the results obtained using MCA requires a detailed study of the mathematical models describing these systems. Thus, it appears that the MCA alone cannot reveal regulatory mechanisms of metabolic systems in the presence of strong allosteric and feedback regulation.

The analysis of subsystem (2), which includes glycolysis and ATPase, using modular metabolic control analysis (MMCA) did not yield fundamentally new results compared with classical MCA. Similar to MCA, this analysis showed that, in a subsystem comprising glycolysis and ATPase, the glycolytic rate is determined mainly by ATPase activity. However, this approach does not provide additional insight into the mechanisms underlying regulation of the glycolytic rate in this subsystem. In our view, a much more informative approach is to analyze the graphs of the steady-state dependencies of ATP production and consumption rates on [ATP], shown in Figure 9. A comparison of these graphs indicates that the intrinsic regulation of glycolytic flux in subsystem (2) is preserved, whereas the inclusion of ATPase imposes an additional constraint that prevents the system from deviating substantially from the steady state. Comparison of the curves in Figure 9 also allows estimation of the degree of ATP concentration stabilization in response to changes in ATPase activity. Moreover, it makes it possible to determine the range of admissible changes in ATPase activity over which glycolytic regulation can ensure an increase in ATP production in accordance with increased ATP consumption. In our earlier studies, we proposed using graphical characteristics of metabolic systems for the analysis of their regulation [15,29,35], but this approach had not been sufficiently developed until now.

## 5. Conclusions

Metabolic control analysis (MCA) was applied to several subsystems derived from the model of human erythrocyte energy metabolism. In subsystems containing only glycolysis and assuming constant concentrations of ATP, ADP, and AMP, MCA correctly identifies the main regulatory steps of the system, namely hexokinase and phosphofructokinase reactions, with control coefficients of 0.838 and 0.149, respectively.

However, in extended subsystems that include glycolysis together with ATPase activity or transmembrane ion transport, the original regulatory mechanisms of glycolysis are no longer clearly revealed in the MCA results. This limitation likely arises because MCA identifies rate-limiting steps rather than true regulatory sites in metabolic systems.

In addition, the primary outputs of MCA are elasticities and control coefficients. These parameters are differential characteristics of the system. They describe system behavior only locally around the given steady state. As a result, MCA may not provide a sufficiently complete description of metabolic system behavior and regulation.

In our opinion, a more informative approach to understanding the behavior and regulation of metabolic systems is the analysis of integral characteristics, such as steady-state dependencies of key metabolite production and consumption rates on its concentration.

## Figures and Tables

**Table 2:** Control coefficient values of glycolytic enzymes, ATPase, Na/K-pump, and cell membrane permeability for cations for glycolytic flux in all subsystems studied in the paper.

Variable Parameter—Activity of Enzyme or Membrane Permeability	Subsystem ^a^						
	(1)	(1a)	(1b)	(2)	(2a)	(2b)	(3)
HK	0.838335	0.838335	0.838346	0.151538	0.001248	1.248470	0.009794
GPI	0.012321	0.012321	0.012347	0.002223	0.000018	0.018380	0.000141
PFK	0.149360	0.149360	0.149322	0.026972	0.000222	0.222316	0.001696
ALD	0 ^b^		0	0	0	0	0
TPI	0		0	0	0	0	0
GAPDH	0		0	0	0	0	−0.000147
PGK	0		0	0	0	0	0
PGM	0		0	0	0	0	−0.000002
ENO	0		0	0	0	0	−0.000122
PK	0		0	0	0	0	−0.000147
LDH	0		0	0	0	0	−0.000002
ATPase				0.819412	0.998514	−0.488747	0.050525
Perm							0.938336
Sum	1.000016	1.000016	1.000015	1.000145	1.000002	1.000419	1.000072

^a^ Description of subsystems: (1) Glycolysis at constant [ATP], [ADP], and [AMP]; (1a) Truncated glycolysis—the three first reactions of glycolysis at constant [ATP], [ADP], and [AMP]; (1b) Glycolysis with disabled allosteric regulation of PFK by AMP at constant [ATP], [ADP], and [AMP]; (2) Glycolysis plus ATPase with the linear dependence of the reaction rate on [ATP]; (2a) Glycolysis plus ATPase with the hyperbolic dependence of the reaction rate on [ATP]; (2b) Glycolysis with disabled allosteric regulation of PFK by AMP plus ATPase with the linear dependence of the reaction rate on [ATP]; (3) Glycolysis plus passive and active transmembrane ion transport. ^b^ The absolute value of the control coefficient is less than 10^−6^.

## Data Availability

The original contributions presented in this study are included in the article/Supplementary Materials. Further inquiries can be directed to the corresponding author.

## Supplementary Materials

The following supporting information can be downloaded at https://www.mdpi.com/article/10.3390/life16030414/s1, Equations (S1)–(S41): description of model definitions and parameters; Figure S1: Effect of changing the system parameters on the profile of glycolytic metabolites in the subsystem (1); Figure S2: Effect of changing the system parameters on the profile of glycolytic metabolites in the subsystem (2); Figure S3: Effect of changing the system parameters on the profile of glycolytic metabolites and ions in the subsystem (3).
