# Supplementary material for: Virtual Cell and Metabolic Control Analysis: Control Coefficients for Glycolytic Flux Are Highly Dependent on the Subsystem Selected for Analysis
Source: Life (Basel). 2026 Mar 4;16(3):414. doi: 10.3390/life16030414 (PMC13027950; doi:10.3390/life16030414)
Supplement: Supplementary file 1 [file life-16-00414-s001.zip › life-4111187-supplementary.pdf]

Virtual Cell and Metabolic Control Analysis: Control Coefficients for Glycolytic Flux Are Highly Dependent on the Subsystem Selected for Analysis

Michael V. Martinov [1], Fazoil I. Ataullakhanov [1], Eugene S. Protasov [1,2] and Victor M. Vitvitsky [1,*]
[1] Center for Theoretical Problems of Physico-Chemical Pharmacology, Russian Academy of Sciences, Moscow 109029, Russia
[2]Dmitriy Rogachev National Medical Research Center for Pediatric Hematology, Oncology, and Immunology, Ministry of Healthcare of the Russian Federation, Moscow 117198, Russia
*Correspondence: victor_vitvitsky@yahoo.com

Supplementary Materials

All equations and parameter values below are based on the data presented in (Martinov et al. 2000)

Differential equations for concentrations of intracellular metabolites and ions used in this study:

$$\frac{d[G6P]}{dt} = V_{HK} - V_{GPI} \quad \text{(S1)}$$

$$\frac{d[F6P]}{dt} = V_{GPI} - V_{PFK} \quad \text{(S2)}$$

$$\frac{d[FDP]}{dt} = V_{PFK} - V_{ALD} \quad \text{(S3)}$$

$$\frac{d[DAP]}{dt} = V_{ALD} - V_{TPI} \quad \text{(S4)}$$

$$\frac{d[GAP]}{dt} = V_{ALD} + V_{TPI} - V_{GAPDH} \quad \text{(S5)}$$

$$\frac{d[1{,}3\text{-}DPG]}{dt} = V_{GAPDH} - V_{PGK} \quad \text{(S6)}$$

$$\frac{d[3\text{-}PG]}{dt} = V_{PGK} - V_{PGM} \quad \text{(S7)}$$

$$\frac{d[2\text{-}PG]}{dt} = V_{PGM} - V_{ENO} \quad \text{(S8)}$$

$$\frac{d[PEP]}{dt} = V_{ENO} - V_{PK} \quad \text{(S9)}$$

$$\frac{d[NAD]}{dt} = V_{LDH} - V_{GAPDH} \quad \text{(S10)}$$

$$\frac{de}{dt} = V_{PGK} + V_{PK} - V_{HK} - V_{PFK} - V_{ATPase} \quad \text{(S11)}$$

$$\frac{de}{dt} = V_{PGK} + V_{PK} - V_{HK} - V_{PFK} - V_{pump} \quad \text{(S12)}$$

$$\frac{d[Na^+]}{dt} = V_{Na} - 3V_{pump} \quad \text{(S13)}$$

$$\frac{d[K^+]}{dt} = V_K + 2V_{pump} \quad \text{(S14)}$$

Algebraic equations used in this study:

$$[NAD] + [NADH] = 0.05\ mM \quad \text{(S15)}$$

$$[ATP] + [ADP] + [AMP] = 1.745 \text{ mM} \quad \text{(S16)}$$

$$\frac{[ADP]^2}{[ATP][AMP]} = 1 \quad \text{(S17)}$$

$$e = 2[ATP] + [ADP] \quad \text{(S18)}$$

$$[K^+]_{int} + [Na^+]_{int} - [A^-]_{int} + ZW = 0 \quad \text{(S19)}$$

$$[K^+]_{int} + [Na^+]_{int} + [A^-]_{int} + W + \omega = 2L \quad \text{(S20)}$$

$$\frac{[A^-]_{ext}}{[A^-]_{int}} = exp\left(-\frac{\Delta\varphi F}{RT}\right) \quad \text{(S21)}$$

[Glucose] = 5 mM (S22)

[Orthophosphate] = 1 mM (S23)

[PYR] = 0.07 mM (S24)

[LAK] = 1.2 mM (S25)

Here $L = 150\ mM$ is a half of physiological blood osmolarity, $W = 48.7\ mM$ is concentration of osmotically active molecules in the cell, excluding adenine nucleotides and metabolites of glycolysis, $Z = -\ 0.593$ is an average charge of such molecules, ω is a sum of concentrations of non-permeating glycolytic metabolites and adenine nucleotides.

Equations included in different subsystems:

Subsystem (1): equations S1 - S10, S15-S17

Subsystem (1a): equations S1 – S3, S16, S17

Subsystem (1b): equations S1 - S10, S15-S17, with disabled activation of PFK by AMP

Subsystem (2): equations S1 - S11, S15 - S18, with linear ATPase

Subsystem (2a): equations S1 - S11, S15 - S18, with hyperbolic ATPase

Subsystem (2b): equations S1 - S11, S15 - S18, with disabled activation of PFK by AMP

Subsystem (3) equations S1 - S10, S12 – S21

Equation S22 was included in all subsystems; equations S23-S25 were included in all subsystems except subsystem (1a).

Equations for the enzymatic reaction rates and passive transmembrane ion fluxes:

<u>Hexokinase (HK)</u>

$$V_{HK} = a_{HK} \frac{\frac{[ATP]}{K_{HK}^1}}{1+\frac{[ATP]}{K_{HK}^1}+\frac{[G6P]}{K_{HK}^2}} \tag{S26}$$

$a_{HK} = 12\ mM/h, K_{HK}^1 = 1\ mM, K_{HK}^2 = 5.5 \cdot 10^{-3}\ mM$

Glucose-6-phosphate isomerase (GPI)

$$V_{GPI} = a_{GPI} \frac{([G6P]-[F6P]K_{GPI}^1)/K_{GPI}^2}{1+\frac{[G6P]}{K_{GPI}^2}+\frac{[F6P]}{K_{GPI}^3}} \tag{S27}$$

$a_{GPI} = 360\ mM/h, K_{GPI}^1 = 3\ mM, K_{GPI}^2 = 0.3\ mM, K_{GPI}^3 = 0.2\ mM$

Phosphofructokinase (PFK)

$$V_{PFK} = a_{PFK} \frac{1.1 \cdot [ATP][F6P]\left(\frac{1}{1+[AMP]/K_{PFK}^3}+\frac{2[AMP]}{K_{PFK}^3+[AMP]}\right)}{(K_{PFK}^2+[ATP])(K_{PFK}^1+[F6P])\left(1+10^8\frac{\left(1+[ATP]/K_{PFK}^4\right)^4}{\left(1+[AMP]/K_{PFK}^3\right)^4\left(1+[F6P]/K_{PFK}^5\right)^4}\right)} \tag{S28}$$

$a_{PFK} = 380\ mM/h, K_{PFK}^1 = 0.1\ mM, K_{PFK}^2 = 2\ mM, K_{PFK}^3 = 0.01\ mM, K_{PFK}^4 = 0.195\ mM,$
$K_{PFK}^5 = 3.7 \cdot 10^{-4}\ mM$

Aldolase (ALD)

$$V_{ALD} = a_{ALD} \frac{\frac{[FDP]}{K_{ALD}^1}-\frac{[DAP][GAP]}{K_{ALD}^2}}{1+\frac{[FDP]}{K_{ALD}^3}+\frac{[DAP]}{K_{ALD}^4}+\frac{[GAP]}{K_{ALD}^5}+\frac{[FDP][DAP]}{K_{ALD}^3 K_{ALD}^4}+\frac{[DAP]^2}{K_{ALD}^4 K_{ALD}^6}+\frac{[DAP][GAP]}{K_{ALD}^4 K_{ALD}^7}} \tag{S29}$$

$a_{ALD} = 76\ mM/h, K_{ALD}^1 = 2 \cdot 10^{-4}\ mM, K_{ALD}^2 = 1.2 \cdot 10^{-5}\ mM^2, K_{ALD}^3 = 0.01\ mM, K_{ALD}^4 =$
$0.032\ mM, K_{ALD}^5 = 2.1 \cdot 10^{-3}\ mM, K_{ALD}^6 = 2\ mM, K_{ALD}^7 = 0.065\ mM$

Triose phosphate isomerae (TPI)

$$V_{TPI} = a_{TPI} \frac{([DAP]-\frac{[GAP]}{K_{TPI}^2})/K_{TPI}^1}{1+\frac{[DAP]}{K_{TPI}^1}+\frac{[GAP]}{K_{TPI}^3}} \tag{S30}$$

$a_{TPI} = 3000\ mM/h, K_{TPI}^1 = 0.82\ mM, K_{TPI}^2 = 0.45\ mM, K_{TPI}^3 = 0.43\ mM$

Glyceraldehyde phosphate dehydrogenase (GAPDH)

$$V_{GAPDH} = a_{GAPDH} \frac{([GAP][NAD][P_i]-[1,3DPG][NADH]/K_{GAPDH}^4)/K_{GAPDH}^1 K_{GAPDH}^2 K_{GAPDH}^3}{1.29\left(1+\frac{[GAP]}{K_{GAPDH}^1}+\frac{[1,2DPG]}{K_{GAPDH}^5}\right)\left(1+\frac{[NAD]}{K_{GAPDH}^2}+\frac{[NADH]}{K_{GAPDH}^6}\right)} \quad (S31)$$

$a_{GAPD} = 690$ mM/h, $K_{GAPD}^1 = 0.13$ mM, $K_{GAPD}^2 = 0.13$ mM, $K_{GAPD}^3 = 3.4$ mM, $K_{GHAPD}^4 = 0.136$ mM, $K_{GAPD}^5 = 0.013$ mM, $K_{GAPD}^6 = 2 \cdot 10^{-3}$

Phosphoglycerate kinase (PGK)

$$V_{PGK} = a_{PGK} \frac{([1,3DPG][ADP]-[3PG][ATP]/K_{PGK}^3)/K_{PGK}^1 K_{PGK}^2}{1+\frac{[ATP]}{K_{PGK}^5}+\frac{[ADP]}{K_{PGK}^2}+\frac{A[1,3DPG]}{K_{PGK}^1}+\frac{B[3PG]}{K_{PGK}^6}} \quad (S32)$$

$A = (K_{PGK}^4 + [ADP] + K_{PGK}^4[ATP]/K_{PGK}^5)/K_{PGK}^2$

$B = (K_{PGK}^7 + [ATP] + K_{PGK}^7[ATP]/K_{PGK}^2)/K_{PGK}^5$

$a_{PGK} = 7330$ mM/h, $K_{PGK}^1 = 2.2 \cdot 10^{-3}$ mM, $K_{PGK}^2 = 0.14$ mM, $K_{PGK}^3 = 380$ mM, $K_{PGK}^4 = 0.3$ mM, $K_{PGK}^5 = 0.27$ mM, $K_{PGK}^6 = 1.4$ mM, $K_{PGK}^7 = 0.4$ mM

Phosphoglycerate mutase (PGM)

$$V_{PGM} = a_{PGM} \frac{([3PG]-[2PG]/K_{PGM}^2)/K_{PGM}^1}{1+\frac{[3PG]}{K_{PGM}^1}+\frac{[2PG]}{K_{PGM}^1}} \quad (S33)$$

$a_{PGM} = 1100$ mM/h, $K_{PGM}^1 = 0.27$ mM, $K_{PGM}^2 = 0.24$, $K_{PGM}^3 = 0.02$ mM

Enolase (ENO)

$$V_{ENO} = a_{ENO} \frac{([2PG]-[PEP]/K_{ENO}^2)/K_{ENO}^1}{1+\frac{[2PG]}{K_{ENO}^1}+\frac{[PEP]}{K_{ENO}^3}} \quad (S34)$$

$a_{ENO} = 83$ mM/h, $K_{ENO}^1 = 0.056$ mM, $K_{ENO}^2 = 6.7$, $K_{ENO}^3 = 2 \cdot 10^{-3}$ mM

Pyruvate kinase (PK)

$$V_{PK} = a_{PK} \frac{[PEP][ADP]/K_{PK}^1 K_{PK}^2}{1+\frac{[ATP]}{K_{PK}^3}+\frac{[ADP]}{K_{PK}^2}+\frac{[PEP]}{K_{PK}^1}+\frac{[PEP][ADP]}{K_{PK}^1 K_{PK}^2}} \quad (S35)$$

$a_{PK} = 120$ mM/h, $K_{PK}^1 = 0.05$ mM, $K_{PK}^2 = 0.43$ mM, $K_{PK}^3 = 0.35$ mM

Lactate dehydrogenase (LDH)

$$V_{LDH} = a_{LDH} \frac{([PYR][NADH] - [LAC][NAD]/K_{LDH}^3)/K_{LDH}^1 K_{LDH}^2}{1 + \frac{[PYR]}{K_{LDH}^1} + \frac{[NADH]K_{LDH}^4 + [PYR][NADH] + [LAC][NADH]K_{LDH}^4/K_{LDH}^5}{K_{LDH}^1 K_{LDH}^2} + \frac{C}{K_{LDH}^5 K_{LDH}^6}} \quad (S36)$$

$$C = K_{LDH}^7[NAD] + K_{LDH}^6[LAC] + [NAD][LAC] + K_{LDH}^7[PYR][NAD]/K_{LDH}^1$$

$a_{LDH} = 550\ mM/h$, $K_{LDH}^1 = 0.022\ mM$, $K_{LDH}^2 = 7 \cdot 10^{-3}\ mM$, $K_{LDH}^3 = 426$, $K_{LDH}^4 = 0.14\ mM$, $K_{LDH}^5 = 380\ mM$, $K_{LDH}^6 = 0.1\ mM$, $K_{LDH}^7 = 170\ mM$

Linear ATPase

$$V_{ATPase}^L = \alpha_{ATPase}^L[ATP] \quad (S37)$$

$\alpha_{ATPase}^L = 0.152\ mM/h$

Hyperbolic ATPase

$$V_{ATPase}^H = \alpha_{ATPase}^H \frac{[ATP]}{[ATP] + K_{ATPasse}} \quad (S38)$$

$a_{ATPase} = 2.255\ mM/h$, $K_{ATPase} = 0.01\ mM$

Na/K- pump, Na/K-ATPase (rate of ATP consumption)

$$V_{pump} = a_{pump}[Na^+][ATP] \quad (S39)$$

$a_{pump} = 0.152\ mM/h$

Passive Na flux through the cell membrane

$$V_{Na} = P_{Na} \frac{\frac{\Delta\varphi F}{RT}}{exp\left(\frac{\Delta\varphi F}{RT}\right) - 1}\left([Na^+]_{ext} - [Na^+]_{int} exp\left(\frac{\Delta\varphi F}{RT}\right)\right) \quad (S40)$$

$P_{Na} = 0.0122$ ч$^{-1}$, $[Na^+]_{ext} = 145\ mM$

Passive K flux through the cell membrane

$$V_K = P_K \frac{\frac{\Delta\varphi F}{RT}}{exp\left(\frac{\Delta\varphi F}{RT}\right) - 1}\left([K^+]_{ext} - [K^+]_{int} exp\left(\frac{\Delta\varphi F}{RT}\right)\right) \quad (S41)$$

$P_K = 0.0124$ ч$^{-1}$, $[K^+]_{ext} = 5\ mM$

Designations and abbreviations:

1,3-DPG – 1,3-biphophoglycerate

2-PG – 2-phophoglycerate

3-PG – 3-phophoglycerate

DAP – dihydroxyacetone phosphate

F6P – fructose-6-phosphate

FDP – fructose 1,6-diphosphate

G6P – glucose-6-phosphate

GAP – glyceraldehyde-3- phosphate

LAC - lactate

PEP - phosphoenolpyruvate

PYR - pyruvate

$\Delta\varphi$ – transmembrane potential

F - Faraday constant

R – molar gas constant

T – temperature

References

Martinov, M.V.; Plotnikov, A.G.; Vitvitsky, V.M.; Ataullakhanov, F.I. Deficiencies of Glycolytic Enzymes as a Possible Cause of Hemolytic Anemia. Biochim. Biophys. Acta **2000**, 1474, 75–87, doi:10.1016/s0304-4165(99)00218-4.

Figures

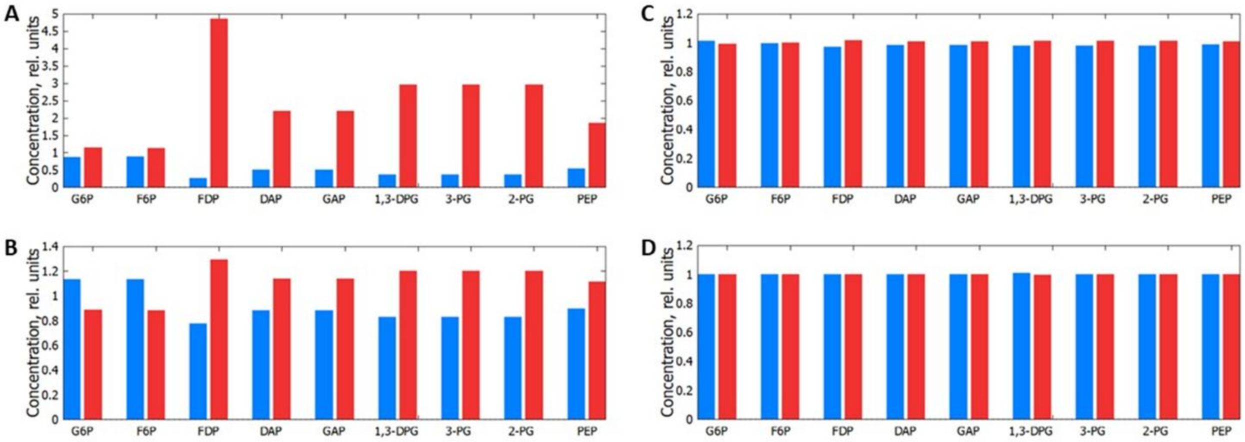

Figure S1. Effect of changing the system parameters on the profile of glycolytic metabolites in the subsystem (1). The varied parameters are A - HK activity, B - PFK activity, C - GPI activity and D - PGK activity. The concentration of each metabolite was normalized to its value obtained with the initial parameter value. Blue and red bars show the relative concentrations of metabolites obtained with a two-fold decrease and a two-fold increase in the parameter value, respectively.

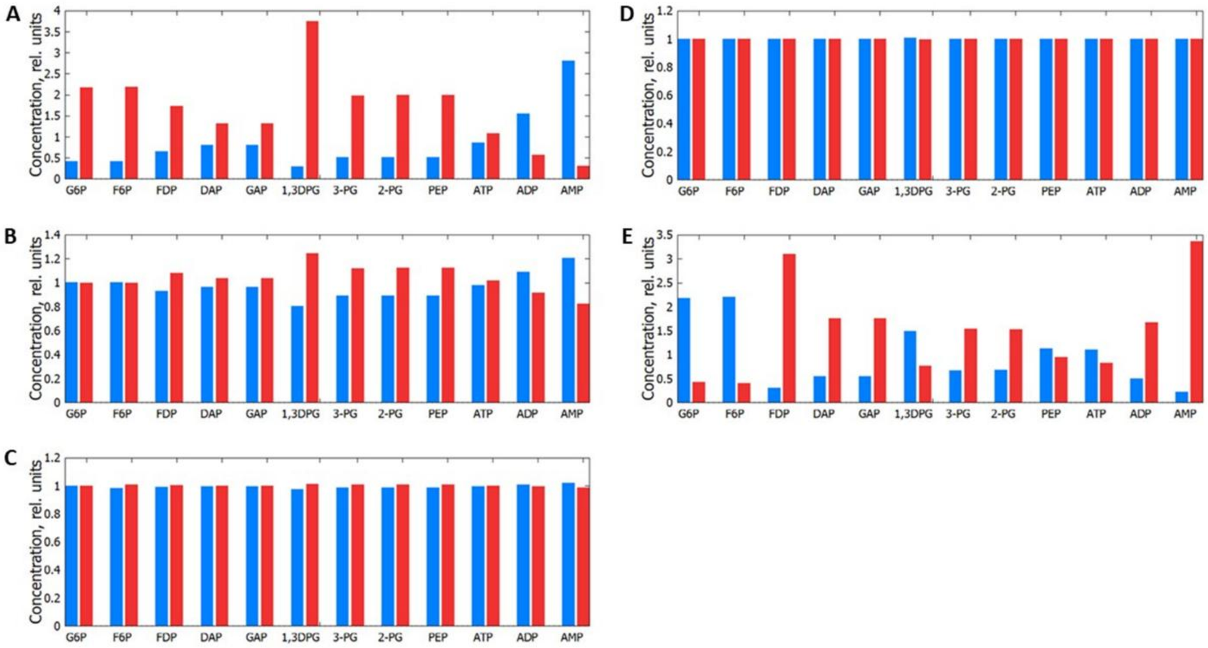

Figure S2. Effect of changing the system parameters on the profile of glycolytic metabolites in the subsystem (2). The varied parameters are A - HK activity, B - PFK activity, C - GPI activity D - PGK activity, and E – ATPase activity. The concentration of each metabolite was normalized to its value obtained with the initial parameter value. Blue and red bars show the relative concentrations of metabolites obtained with a two-fold decrease and a two-fold increase in the parameter value, respectively.

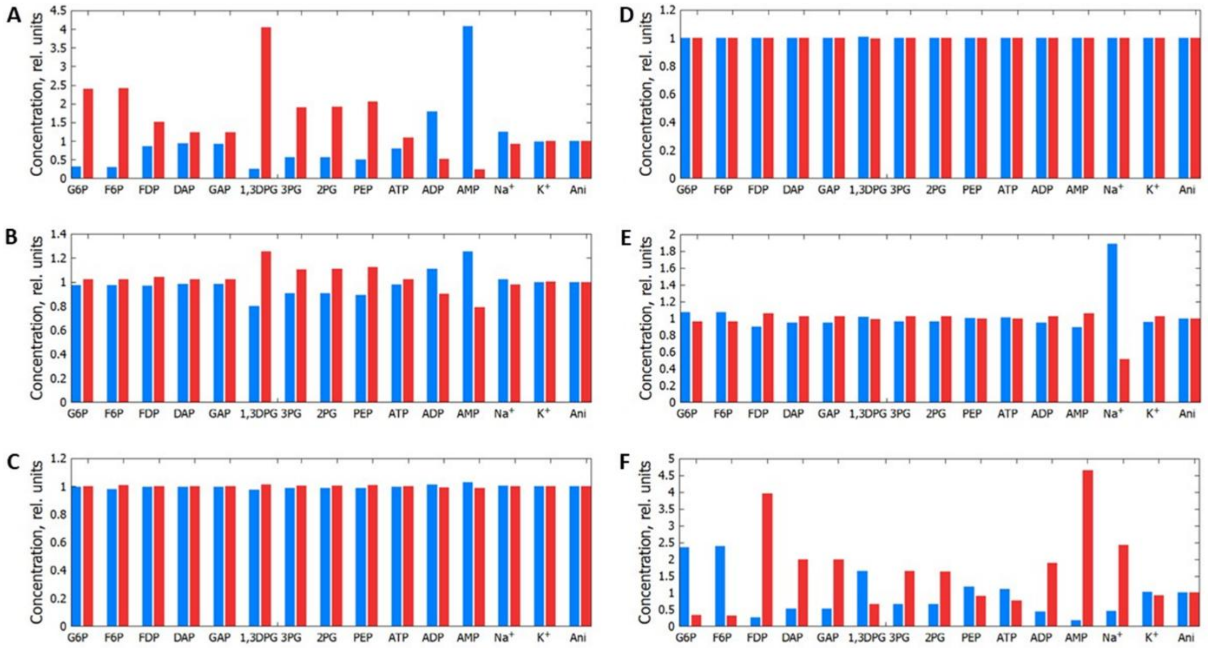

Figure S3. Effect of changing the system parameters on the profile of glycolytic metabolites and ions in the subsystem (3). The varied parameters are A - HK activity, B - PFK activity, C - GPI activity, D - PGK activity, E – Na/K-ATPase activity, and F – the cell membrane permeability to cations. The concentration of each metabolite or ion was normalized to its value obtained with the initial parameter value. Blue and red bars show the relative concentrations of metabolites and ions obtained with a two-fold decrease and a two-fold increase in the parameter value, respectively.
